# Association between timing and consistency of physical activity and type 2 diabetes: a cohort study on participants of the UK Biobank

**DOI:** 10.1007/s00125-023-06001-7

**Published:** 2023-09-20

**Authors:** Caiwei Tian, Charlyne Bürki, Kenneth E. Westerman, Chirag J. Patel

**Affiliations:** 1 Faculty of Arts and Sciences, Harvard University, Cambridge, MA USA; 2 Department of Biomedical Informatics, Harvard Medical School, Boston, MA USA; 3 Swiss Federal Institute of Technology Lausanne, Lausanne, Switzerland; 4 Clinical and Translational Epidemiology Unit, Mongan Institute, Massachusetts General Hospital, Boston, MA USA; 5 Department of Medicine, Harvard Medical School, Boston, MA USA; 6 Programs in Metabolism and Medical and Population Genetics, Broad Institute of MIT and Harvard, Cambridge, MA USA

**Keywords:** Exercise routine, Lifestyle factors, Physical activity, Type 2 diabetes

## Abstract

**Aims/hypothesis:**

We sought to quantify the relationship between morning, afternoon or evening physical activity and consistency (e.g. routine) and risk of type 2 diabetes.

**Methods:**

A cohort of 93,095 UK Biobank participants (mean age 62 years) without a history of type 2 diabetes wore a wrist-worn accelerometer for 1 week. We converted accelerometer information to estimate metabolic equivalent of task (MET), summing MET h of total physical activity completed within three intra-day time segments (morning, afternoon and evening). We quantified physical activity consistency as the SD of participants’ daily total physical activity. We ultimately associated each of the following with incident type 2 diabetes: (1) morning, afternoon or evening ‘time-segmented’ MET h per week; and (2) consistency. We also considered moderate-to-vigorous physical activity (MVPA) and vigorous physical activity (VPA) in association with type 2 diabetes incidence.

**Results:**

When considering MET as the physical activity measure, we observed protective associations of morning (HR 0.90 [95% CI 0.86, 0.93],* p*=7×10^−8^) and afternoon (HR 0.91 [95% CI 0.87, 0.95], *p*=1×10^−5^) but did not have evidence for evening physical activity (HR 0.95 [95% CI 0.90, 1.00], *p*=0.07) with type 2 diabetes. There was no difference between MET-measured morning and afternoon physical activity. Our substitution model highlighted the importance of adjusting for lifestyle factors (e.g. sleep time and diet); the effect of a substitution between afternoon and evening physical activity was attenuated after adjustment for lifestyle variables. Consistency of MET-measured physical activity was not associated with type 2 diabetes (*p*=0.07). MVPA and VPA were associated with decreased risk for type 2 diabetes at all times of the day.

**Conclusions/interpretation:**

Total metabolic equivalents of physical activity in the morning and afternoon had a protective effect on diabetes risk and evening activity was not associated with diabetes. Consistency of physical activity did not play a role in decreasing risk for diabetes. Vigorous activity is associated with lower risk no matter the time of day of activity.

**Graphical Abstract:**

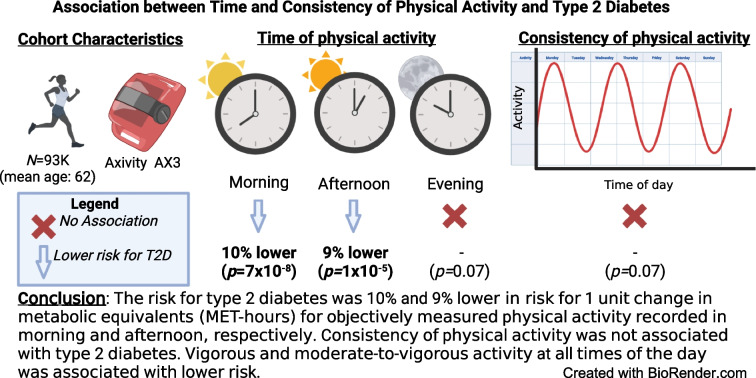

**Supplementary Information:**

The online version contains peer-reviewed but unedited supplementary material available at 10.1007/s00125-023-06001-7.


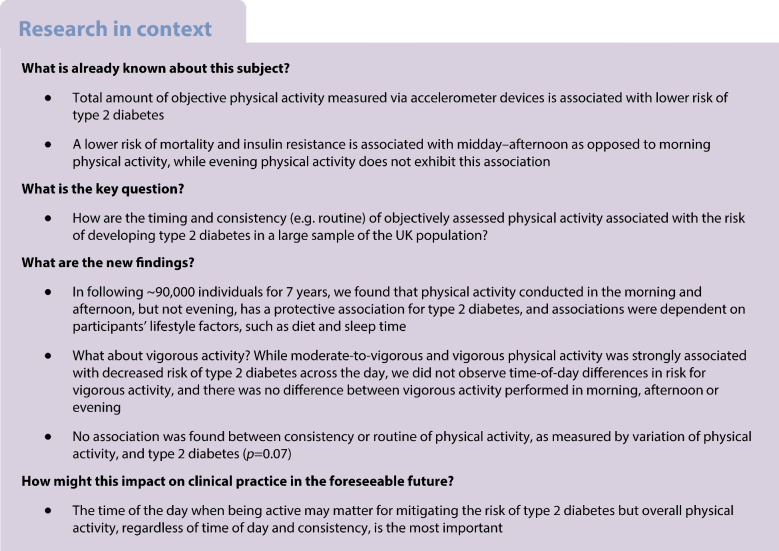


## Introduction

Physical activity is a preventive factor for type 2 diabetes but its timing and consistency (in contrast with overall sum of physical activity) has been relatively unexplored. It has been shown that midday–afternoon but not evening physical activity is associated with lower risk of mortality compared with morning physical activity but the relationship with type 2 diabetes remains understudied [1]. We sought to assess the relationship between objectively measured physical activity timing and consistency (e.g. routine) and risk of type 2 diabetes. We aimed to avoid recall biases by analysing total, type, duration and consistency of objectively measured physical activity via an accelerometer. Secondarily, we sought to assess whether moderate-to-vigorous physical activity (MVPA) or vigorous physical activity (VPA) were associated with decreased risk for type 2 diabetes for activity in morning, afternoon or evening.

## Methods

The UK Biobank is a cohort with 502,664 adults aged between 37 and 73 years at baseline, recruited between 2006 and 2010 from England, Scotland and Wales. Health and lifestyle information was collected by both self-reported questionnaires and measurement at assessment centres. From 2013 to 2015, wrist-worn accelerometers (Axivity AX3; Axivity, Newcastle, UK [https://axivity.com/product/ax3]) were given randomly to 103,686 UK Biobank participants, who were asked to wear the device on their dominant wrist for a continuous period of 7 days. The raw accelerometer data were pre-processed by the Biobank Accelerometer Analysis software (https://github.com/OxWearables/biobankAccelerometerAnalysis, accessed on 1 May 2021) followed by metabolic equivalent of task (MET) calculation as described by Doherty et al and Le Goallec et al [2, 3]. We removed participants with less than seven full days of data.

MET is the rate of energy a participant expends performing a task compared with when they are sitting or standing still (being inactive) [4]. The resting MET is set at 1. The MET h unit measures the total energy expenditure over 1 h. For example, if a participant performs a task with a MET of 2 for 3 h, then the total energy expenditure for this participant equals 2×3 = 6 MET h. A typical amount of activity over a 24 h period is 43.4 MET h (median among UK Biobank participants). We divided the day’s typical waking hours into three blocks of 6 h: 06:00–12:00 hours (morning); 12:00–18:00 hours (afternoon); and 18:00–24:00 hours (evening). We calculated the total amount of activity for each participant and time block during the week-long period of accelerometer usage, as previously described by van der Velde et al [5]. Next, we computed the SD of daily MET h throughout the week as a measure of total physical activity consistency. Hospital records for type 2 diabetes information, encoded as E11 according to ICD-10 (http://apps.who.int/classifications/icd10/browse/2016/en), were available until February 2021 in England and Scotland and until February 2018 in Wales. For people who died, we counted type 2 diabetes diagnosis date as an event, whereas death was considered a censoring non-event. We removed participants who were diagnosed with type 2 diabetes at baseline assessment or those who developed type 2 diabetes within 1 year of their physical activity measurement to reduce reverse causation. We also excluded those whose baseline HbA_1c_ was greater than 48 mmol/mol (6.5%; a recommended cutoff point for diagnosing type 2 diabetes) [6].

We tested the association of MET h with incident type 2 diabetes using a Cox proportional hazards model. We modelled the relationship between total MET h and incident type 2 diabetes as linear after seeing no improvement in model fit using a cubic spline (assessed by a likelihood ratio test [LRT]). To test for the associations of time-specific activity, we fitted a Cox model with separate terms for morning, afternoon and evening MET h, adjusting for age, sex, BMI, ethnicity, socioeconomic factors including education, household income before tax, and lifestyle factors, such as smoking status, alcohol intake, sleep duration, wake time and diet. In particular, the dietary information encompassed vegetable and fruit intake, meat and fish consumption, and bread type (grain or not) eaten by the study participants. Time and age were measured in years in the Cox model.

We estimated the effect of physical activity timing substitution (substituting a MET h of physical activity at one time with another time) using the effect estimates and covariances from the timing Cox model. We fitted three sets of models in the substitution analysis: (1) only adjusting for basic information (sex, age, ethnicity and BMI); (2) adding adjustment for socioeconomic factors (education and household income); and (3) adding adjustment for lifestyle factors (smoking status, alcohol intake, sleep duration and dietary information). We also examined the associations stratified by sex and age (individuals younger or older than the age of 65 years).

We also examined the association between MVPA and VPA and type 2 diabetes. We tested for improved model fit using time-specific rather than overall summed activity using an LRT. Finally, to test the association between total physical activity routine and type 2 diabetes, we fitted a separate model testing an individual-level SD of daily MET h over a week, adjusting for total weekly MET h and sex, age, ethnicity, BMI, education, household income, smoking status, alcohol intake, sleep duration and dietary information.

## Results

**Fig. 1:**
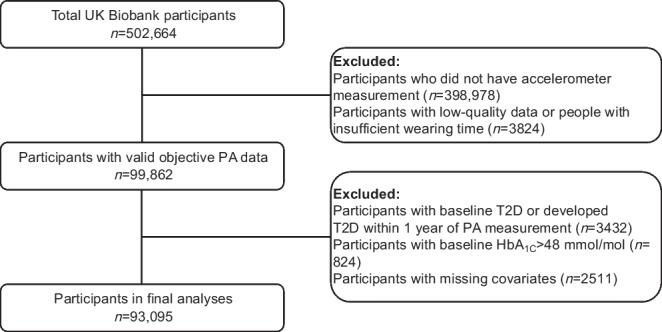
Flow diagram of participants included in the study. PA, physical activity; T2D, type 2 diabetes

There were 93,095 participants in our study, with a mean of 6.64 follow-up years and SD of 0.77 (IQR 6–7 [range 2–8] years) (Fig. 1). Population characteristics are shown in Table 1. We observed that participants in our sample were older and had higher household income, and were more likely to have smoked and have a lower baseline HbA_1c_ compared with the entire UK Biobank cohort (electronic supplementary material [ESM] Table 1). We analysed the Schoenfeld residuals to test the proportionality assumption of the Cox model and concluded that our models did not violate the proportional hazards assumption and that the HRs were constant through follow-up time.

**Table 1:** Participant characteristics

Characteristic	Overall	Most PA in the morning	Most PA in the afternoon	Most PA in the evening
Count	93,095 (100)	13,633 (15)	76,530 (82)	2932 (3)
Age, years	61.9±8.1	62.2±8.3	62.0±8.0	56.4±7.5
Age ≤65 years	56,535 (61.4)	7906 (58)	46,125 (60.3)	2504 (85.4)
Age >65 years	36,560 (38.6)	5727 (42)	30,405 (39.7)	428 (14.6)
Sex				
Male	40,387 (43.4)	7128 (52.3)	31,774 (41.5)	1485 (50.6)
Female	52,708 (56.6)	6505 (47.7)	44,756 (58.5)	1447 (49.4)
BMI, kg/m^2^	26.5±4.4	26.7±4.4	26.5±4.3	26.3±4.7
Baseline HbA_1c_, mmol/mol	32.6±8.9	32.7±9.0	32.6±8.9	32.2±9.0
Baseline HbA_1c_, %	5.1±3.0	5.1±3.0	5.1±3.0	5.1±3.0
Sleep time, h	10.08±1.68	10.08±1.92	10.08±1.68	9.36±1.92
Wake time, h^a^	7±1.8	6±2.1	7±1.7	8±2.5
Time spent in education, years	16.4±4.5	16.0±4.7	16.4±4.5	17.5±3.9
Total household income (£) before tax				
<18,000	11,861 (12.7)	1982 (14.5)	9602 (12.5)	277 (9.4)
18,000–30,999	19,943 (21.4)	2971 (21.7)	16,502 (21.5)	470 (16.0)
31,000–51,999	24,398 (26.2)	3402 (24.9)	20,235 (26.4)	761 (26.0)
52,000–100,000	21,753 (23.3)	2962 (21.7)	17,896 (23.3)	895 (30.5)
>100,000	6312 (6.8)	1013 (7.4)	4970 (6.5)	329 (11.2)
Not known	8828 (9.5)	1303 (9.6)	7325 (9.6)	200 (6.8)
Ethnicity (self-reported)				
White	85,791 (92.0)	12,533 (92.0)	70,757 (92.0)	2501 (85.0)
Mixed	2540 (2.7)	368 (2.7)	2054 (2.7)	118 (4.0)
Asian	3494 (3.8)	533 (3.9)	2759 (3.6)	202 (6.9)
Black	328 (0.4)	73 (0.5)	230 (0.3)	25 (0.9)
Chinese	215 (0.2)	20 (0.1)	158 (0.2)	37 (1.3)
Other	727 (0.8)	106 (0.8)	572 (0.7)	49 (1.7)
Smoking status				
Ever smoked	55,527 (59.6)	8243 (60.5)	45,537 (59.5)	1747 (59.6)
Never smoked	37,377 (40.1)	5361 (39.3)	30,833 (40.3)	1183 (40.3)
Not known	191 (0.21)	29 (0.21)	160 (0.21)	2 (0.07)
Alcohol intake				
Daily or almost daily	21,444 (23.3)	3021 (22.2)	17,761 (23.2)	662 (22.6)
Three or four times a week	24,639 (26.4)	3371 (24.7)	20,560 (26.9)	708 (24.1)
Once or twice a week	23,476 (25.2)	3467 (25.4)	19,315 (25.2)	694 (23.7)
One to three times a month	10,079 (10.8)	1413 (10.3)	8296 (10.8)	370 (12.6)
Special occasions only	8523 (9.2)	1448 (10.6)	6769 (8.8)	306 (10.4)
Never	4898 (5.3)	907 (6.7)	3800 (5.0)	191 (6.5)
Not known	36 (0.03)	6 (0.04)	29 (0.04)	1 (0.03)
Self-reported nutrition (serving)				
Weekly fish	2.2 (1.5)	2.2 (1.6)	2.2 (1.5)	2.1 (1.6)
Weekly meat	3.5 (2.1)	3.5 (2.2)	3.4 (2.1)	3.5 (2.4)
Weekly vegetables	4.8 (3.1)	4.9 (3.2)	2.8 (3.1)	4.8 (3.3)
Weekly fruit	3.1 (2.5)	3.3 (2.6)	3.1 (2.4)	2.9 (2.5)
MET h				
Overall (per week)	304.8±24.0	303.9±25.7	304.8±23.5	308.2±26.6
Morning (per day)	12.3±1.6	13.8±1.5	12.0±1.5	11.1±1.6
Afternoon (per day)	13.8±1.5	12.9±1.4	14.1±1.4	12.5±1.4
Evening (per day)	11.2±1.5	10.4±1.4	11.2±1.4	13.3±1.4
MVPA time, h				
Overall (per week)	12.5±5.5	12.7±5.8	12.5±5.4	12.6±5.7
Morning (per day)	0.67±0.3	0.87±0.4	0.62±0.3	0.50±0.3
Afternoon (per day)	0.73±0.4	0.59±0.3	0.76±0.4	0.55±0.3
Evening (per day)	0.36±0.2	0.31±0.2	0.36±0.2	0.65±0.3
VPA time, min				
Overall (per week)	27.91±40.3	31.53±48.5	27.03±38.3	34.20±47.4
Morning (per day)	1.46±3.2	2.37±4.9	1.30±2.7	1.20±2.6
Afternoon (per day)	1.15±2.5	0.82±2.0	1.22±2.6	0.91±2.2
Evening (per day)	0.61±1.8	0.54±1.8	0.57±1.7	1.88±3.7

Data are presented as *n* (%) or mean ± SD

^a^Measured in hours of the day (e.g. 7 means 07:00 hours)

PA, physical activity

Both morning and afternoon physical activity were associated with reduced risk of type 2 diabetes. Each additional MET h of physical activity in the morning was associated with 10% risk reduction of type 2 diabetes (Table 2). Physical activity in the afternoon was associated with similar 9% risk reduction. Evening physical activity was associated with a non-significant 5% reduced type 2 diabetes risk. A model with separate terms corresponding to morning, afternoon and evening MET h explained the variance of type 2 diabetes better than the model using total MET h.

**Table 2:** HR (95% CI) for physical activity by morning, afternoon and evening MET h

Group	Morning physical activity		Afternoon physical activity		Evening physical activity	
	HR (95% CI) per MET h	*p* value	HR (95% CI) per MET h	*p* value	HR (95% CI) per MET h	*p* value
Overall	0.90 (0.86, 0.93)	7×10^−8^	0.91 (0.87, 0.95)	1×10^−5^	0.95 (0.90, 1.00)	0.0698
Male sex	0.90 (0.85, 0.94)	3×10^−5^	0.93 (0.88, 0.98)	0.0064	0.93 (0.87, 1.00)	0.0557
Female sex	0.89 (0.84, 0.95)	0.0003	0.89 (0.83, 0.96)	0.0017	0.98 (0.91, 1.06)	0.6252
Age ≤65 years	0.88 (0.83, 0.93)	3×10^−6^	0.91 (0.87, 0.99)	0.0277	0.87 (0.81, 0.94)	0.0002
Age >65 years	0.92 (0.87, 0.97)	0.0029	0.88 (0.83, 0.93)	3×10^−5^	1.01 (0.98, 1.13)	0.1918

Data are from model set 3, adjusted for sex, age, ethnicity, BMI, education, household income, smoking status, alcohol intake, sleep duration and dietary information

We found that participants who performed moderate levels of morning physical activity (i.e. participants in Q2 and Q3) had 24% and 34% reduction in risk of type 2 diabetes compared with participants who performed little to no morning physical activity (Q1) (ESM Table 2). For those who performed the most morning physical activity (Q5), we found a 38% reduced risk of type 2 diabetes compared with Q1. Participants who were in Q5 of afternoon and evening physical activity had 27% and 24%, respectively, reduced risk compared with participants in Q1.

MVPA and VPA were associated with reduced risk of type 2 diabetes across all times (HRs ≤0.78 for MVPA in hours and HRs ≤0.93 for VPA in minutes), but morning, afternoon and evening risk was dependent on sociodemographic and lifestyle adjustment factors (ESM Table 3). The distribution of MVPA (measured in hours) and VPA (measured in minutes) are shown in ESM Figs 1, 2.

Furthermore, in substitution analyses, the substitution between morning and afternoon was not statistically significant for any of the models (ESM Table 4), reiterating lack of difference between the risks estimated in morning and afternoon. The substitution analyses also demonstrated the sensitivity of the associations to model and lifestyle adjustment variables. A replacement of one MET h of evening physical activity with an equivalent amount in the morning and afternoon was associated with a 7% (95% CI 2%, 11%, *p*=0.0041) and 10% (95% CI 3%, 16%, *p*=0.0036) reduction in type 2 diabetes risk, respectively, for model set 1 (adjusting for age, sex, ethnicity and BMI). For model set 2 (adjusting for variables in model set 1 plus education and household income), substituting one MET h of evening physical activity with an equivalent amount in the morning was associated with 9% reduction in risk (95% CI 4%, 13%, *p*=0.0002); an equivalent amount in the afternoon was associated with 13% reduction in type 2 diabetes risk (95% CI 6%, 19%, *p*=0.0002). The morning–evening substitution for model set 3 (adjusting for variables in model set 2 plus smoking, alcohol intake, sleep duration and dietary information) was associated with 6% risk reduction (95% CI 1%, 11%, *p*=0.0345) but afternoon–evening substitution was not associated with risk reduction (*p*=0.23) for model set 3.

In exploratory analysis, we found modest evidence for physical activity timing effect modification by both sex (*p*=0.02 for evening physical activity) and age (*p*=0.02 for evening physical activity). Women had a small relative benefit from afternoon physical activity when compared with men. For participants in all subgroups, morning and afternoon physical activity were associated with reduced type 2 diabetes risk. Evening physical activity, however, was not associated with risk reduction for female participants or participants aged over 65 years (Table 2).

**Fig. 2:**
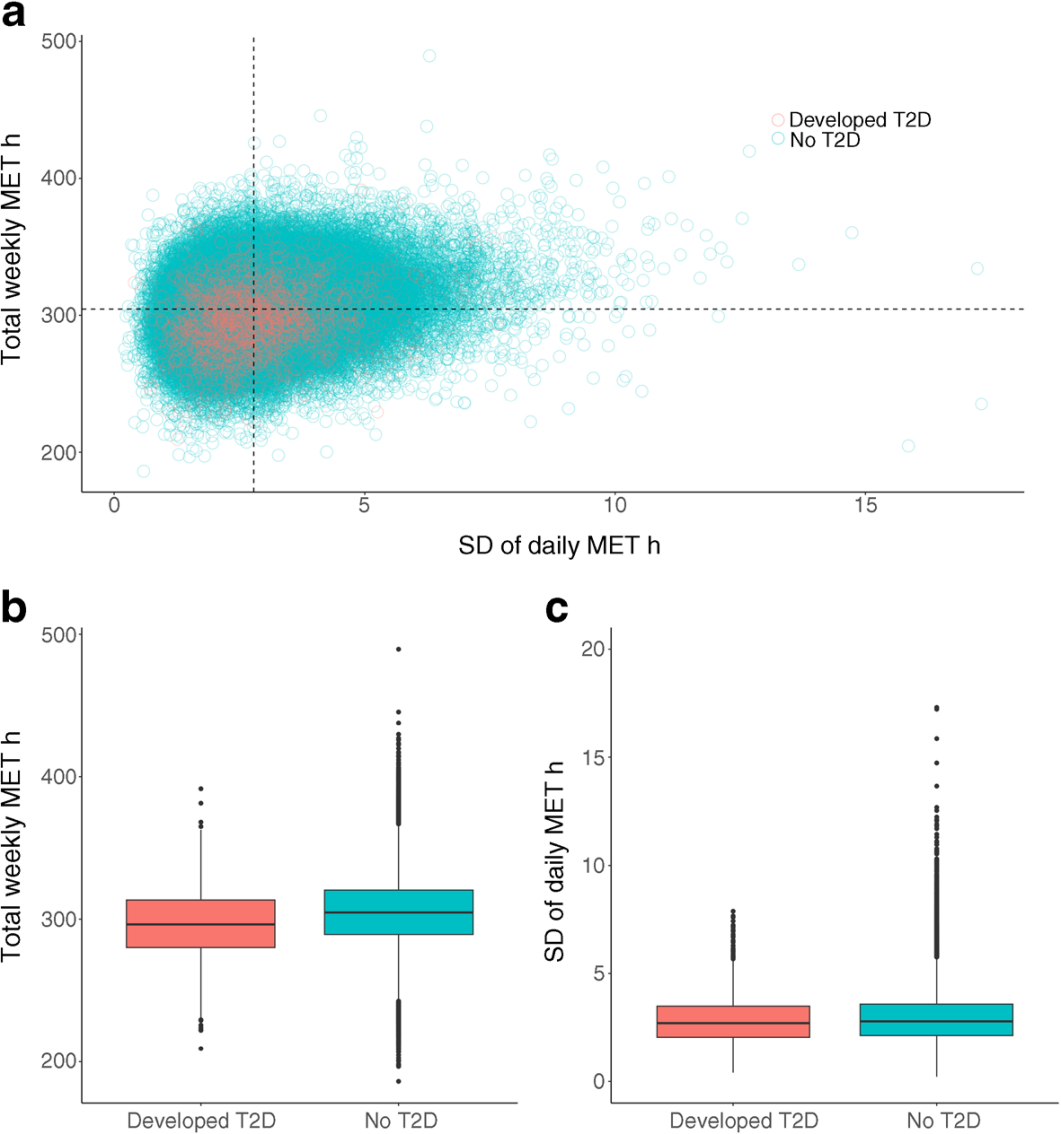
Distribution of within-person daily MET h SD and total weekly MET h for participants who developed type 2 diabetes (*n*=1657) and did not develop type 2 diabetes (*n*=91,438). (**a**) Scatter plot of total weekly MET h vs SD of daily MET h for the two groups. (**b**) Box plots showing the distribution of total weekly MET h for the two groups. (**c**) Box plots showing the distribution of daily MET h SD for the two groups. In the box plots, horizontal lines depict the IQR and median (25th, 50th and 75th percentile). The whiskers represent the min. and max. values and the dots represent outliers. T2D, type 2 diabetes

Compared with participants who developed type 2 diabetes, participants who did not develop type 2 diabetes had higher total weekly MET h and similar daily MET h SD (Fig. 2). The HR for SD of daily MET h (per one unit increase in SD of MET h) was 1.04 (95% CI 1.00, 1.09, *p*=0.068), demonstrating no association between the consistency of total physical activity and type 2 diabetes.

## Discussion

Morning and afternoon physical activity measured by MET h were associated with 9 and 10 per cent lower risk of type 2 diabetes across sociodemographic factors. We found no evidence for association between evening physical activity and type 2 diabetes.

There was a correlation between physical activity behaviour and other attributes of lifestyle, as reflected by the changes in association in timing of physical activity and type 2 diabetes incidence when adjusting for different variables in the model, such as sleep and dietary variables. In addition, while overall MET h are associated with a lower risk for type 2 diabetes, the consistency or routine of physical activity was not strongly associated with type 2 diabetes. In other words, individuals who exercise a smaller amount of time more frequently are at no lesser risk for diabetes than individuals who exercise the same total amount, but with less of a routine.

Our results emphasise the importance of consideration and adjustment for sleep and diet when studying timing of physical activity. For example, when adjusting for sleep and diet (along with education and income), associations for morning MET h became larger and more precise (e.g. HR of 0.90 vs 0.95 when not adjusting for sleep, diet, education and income), and afternoon MET h become more precise. Second, we observed that the type of objective physical activity also matters in testing associations (e.g. MVPA or VPA vs total MET h). Lifestyle has less of an impact on the association of VPA with diabetes. Adding lifestyle factors to the VPA models did not make a sizable difference in the substitution models for morning, afternoon and evening associations (ESM Table 4).

It is hypothesised that physical activity may improve insulin sensitivity and assist in lowering elevated blood glucose levels [5, 7]. Previous investigations have tested the relationship between health and timing of physical activity using different methods of assessment (e.g. count of physical activity, high-intensity interval training, etc.) among specific groups of people at risk for diabetes (e.g. older women, a smaller younger group with 125 participants, men with diabetes, or men with impaired glucose metabolism) [8–11]. Two papers [8, 9] reported physical activity ‘count’ as the measure of choice; the others [10, 11] compared groups of people who did exercise ‘training’ in the morning vs in the afternoon for example. Some have suggested more exercise in the morning, while others have reported afternoon physical activity to be more beneficial. In our study, morning and afternoon activity was shown to both be beneficial, while evening activity was relatively weak in association with diabetes. We believe this is consistent with the result from a similar recent study of mortality [1]; however, we emphasise there were minimal differences in association sizes between the timing groups. The different associations among these studies [1, 8–11] are likely due in part to the differences between physical activity measurements, different population characteristics, and as we also show, choice of lifestyle adjustment variables. In our analyses, we used the MET h as the objective physical activity measurement to take all daily activities into account and, in secondary analyses, showed that the MVPA or VPA modes of activity are associated with decreased risk no matter the time of day. Future investigations should consider types of physical activity (e.g. aerobic vs anaerobic), and assessment of physical activity in association with cardiometabolic outcomes.

We are also, to our knowledge, the first to report that there is no relationship between the consistency of physical activity and type 2 diabetes risk. On the other hand, our findings are in line with other papers showing that the total amount of physical activity is associated with type 2 diabetes [12–14]. Limitations of our study include, first, that the UK Biobank is not representative of the full UK population [15]. Second, while we followed an established method for time-based segmentation, different approaches for characterising ‘morning’, ‘afternoon’ and ‘evening’ times of the day might produce different results [1, 5]. Further, while we did adjust for baseline sociodemographics and behaviours, as well as stratifying our models by age, residual confounding based on socioeconomic status and amount or type of employment might affect the types of individuals that are able to exercise in the morning or afternoon and thus bias the reported associations. Medication is another key variable worth further exploration for its potential to modify the relationship between physical activity timing and type 2 diabetes and its complications. Taken together, our findings support that total physical activity but not its consistency over the week may be an important factor impacting type 2 diabetes risk. Timing of activity may play a role in mitigation of diabetes risk, but difference in risk between time of intervals is small.

### Supplementary Information

Below is the link to the electronic supplementary material.

Supplementary file1 (PDF 127 KB)

## Data Availability

UK Biobank data are available through a procedure described at http://www.ukbiobank.ac.uk/using-the-resource/.

## Abbreviations

LRT: Likelihood ratio test
MET: Metabolic equivalent of task
MVPA: Moderate-to-vigorous physical activity
VPA: Vigorous physical activity
