# Supplementary material for: Association between timing and consistency of physical activity and type 2 diabetes: a cohort study on participants of the UK Biobank
Source: Diabetologia. 2023 Sep 20;66(12):2275–82. doi: 10.1007/s00125-023-06001-7 (PMC10628002; doi:10.1007/s00125-023-06001-7)

Electronic Supplementary Material

Tian et al, Association between Time and Consistency of Physical Activity and Type 2 Diabetes - A Cohort Study on Participants of the UK Biobank

ESM Table 1. Comparison between all UK Biobank participants and subsample in our study

	**Overall***	**Sample in the study**
**Count**	502,409	93,095
**Age mean (std)**	56.5 (8.1)	61.9 (8.1)
Age <= 65	428,543 (85%)	56,535 (61%)
Age > 65	73,866 (15%)	35,560 (39%)
**Sex**		
Male	229,085 (46%)	40,387 (43%)
Female	273,324 (54%)	52,708 (57%)
**BMI mean (std)**	27.4 (4.8)	26.5 (4.4)
**Baseline HbA1C* mean (std)**	36.1 (6.8)	32.6 (8.9)
**Total household income before tax**		
Less than 18,000	97,180 (20%)	11,861 (13%)

18,000 to 30,999	108,157 (22%)	19,943 (21%)
31,000 to 51,999	110,751 (22%)	24,398 (26%)
52,000 to 100,000	86,250 (17%)	21,753 (23%)
Greater than 100,000	22,927 (4.6%)	6,312(6.8%)
Not know	77,144 (15%)	8,828 (9.5%)
**Ethnicity (self-reported)**		
White	472,609 (95%)	85,791 (92%)
Other	29,800 (5%)	7,304 (8%)
**Smoking status**		
Ever smoked	225,986 (46%)	55,527 (60%)
Never smoked	273,473 (55%)	37,377 (40%)
Not know	2,057 (0.4%)	191 (0.2%)
**Alcohol intake**		
Daily or almost daily	101,753 (20%)	21,444 (23%)
Three or four times a week	115,422 (23%)	24,639 (26%)
Once or twice a week	129,269 (26%)	23,476 (25%)
One to three times a month	55,840 (11%)	10,079 (11%)
Special occasions only	57,996 (12%)	8,523(9.2%)
Never	40,627 (8.1%)	4,898 (5.3%)

Not know	1,502 (0.3%)	36 (<0.1%)
**Self-reported nutrition (serving)**		
Weekly fish	2.3 (1.6)	2.2 (1.5)
Weekly meat	3.6 (2.2)	3.5 (2.1)
Weekly vegetables	4.9 (3.4)	4.8 (3.1)
Weekly fruits	3.1 (2.6)	3.1 (2.5)

* The overall population with non-missing genetic and T2D data and available questionnaire responses data

ESM Table 2. Hazard Ratio compared to Q1 using quintile-based regression analyses for PA in the morning, afternoon and evening separately with MET hour as the measurement.

		HR	95% CI	p-value
Morning MET	Q2	0.76	[0.66, 0.87]	0.0001
	Q3	0.66	[0.56, 0.77]	1e-7
	Q4	0.67	[0.56, 0.79]	1e-6
	Q5	0.62	[0.51, 0.75]	4e-7
Afternoon MET	Q2	0.97	[0.85, 1.12]	0.7052
	Q3	0.84	[0.72, 0.99]	0.0338
	Q4	0.79	[0.66, 0.94]	0.0077
	Q5	0.73	[0.60, 0.89]	0.0020
Evening MET	Q2	0.82	[0.71, 0.96]	0.0109
	Q3	0.87	[0.73, 1.03]	0.0956
	Q4	0.87	[0.72, 1.04]	0.1340
	Q5	0.76	[0.61, 0.94]	0.0131

ESM Table 3. Comparison of Hazard Ratio (HR) for PA in the morning, afternoon, and evening, using different PA metrics. MET and MVPA are measured in hours and VPA is measured in minutes.

		Morning			Afternoon			Evening		
PA Metric	Model Set*	HR	95% CI	*p*-value	HR	95% CI	*p*-value	HR	95% CI	*p*-value
MET (hour)	Set 1	0.95	[0.91, 0.98]	0.0011	0.91	[0.87, 0.95]	2e-5	1.01	[0.97, 1.06]	0.4799
	Set 2	0.94	[0.91, 0.98]	0.0009	0.90	[0.86, 0.94]	2e-6	1.03	[0.99, 1.08]	0.0949
	Set 3	0.90	[0.86, 0.93]	7e-8	0.91	[0.87, 0.95]	1e-5	0.95	[0.90, 1.00]	0.0698
MVPA (hour)	Set 1	0.65	[0.53, 0.81]	0.0001	0.75	[0.60, 0.94]	0.0108	0.66	[0.47, 0.93]	0.0192
	Set 2	0.63	[0.51, 0.78]	1e-5	0.74	[0.59, 0.93]	0.0084	0.73	[0.52, 1.03]	0.0774
	Set 3	0.60	[0.48, 0.75]	7e-6	0.78	[0.63, 0.98]	0.0315	0.68	[0.48, 0.96]	0.0290

VPA (minute)	Set 1	0.93	[0.90, 0.96]	8e-5	0.90	[0.87, 0.95]	1e-5	0.91	[0.86, 0.97]	0.0059
	Set 2	0.93	[0.90, 0.96]	9e-5	0.90	[0.86, 0.94]	7e-6	0.92	[0.86, 0.98]	0.0091
	Set 3	0.93	[0.90, 0.97]	0.0002	0.91	[0.87, 0.95]	1e-5	0.92	[0.86, 0.98]	0.0085

* The first set was adjusted for basic information: sex, age, ethnicity and BMI. The second set was added adjustment for socioeconomic factors: education and household income. The third set was further adjusted for lifestyle factors: smoke status, alcohol intake, sleep duration, and dietary information.

ESM Table 4. Effect of substituting PA at different times. MET and MVPA are measured in hours while VPA is measured in minutes.

		SAM[1]			SAE			SME		
PA Measure	Model Set[2]	HR	95% CI	*p*-value	HR	95% CI	*p*-value	HR	95% CI	*p*-value
MET (hour)	Set 1	0.96	[0.90, 1.03]	0.2829	0.90	[0.84, 0.97]	0.0036	0.93	[0.89, 0.98]	0.0041
	Set 2	0.96	[0.90, 1.02]	0.1689	0.87	[0.81, 0.94]	0.0002	0.91	[0.87, 0.96]	0.0002
	Set 3	1.01	[0.94, 1.08]	0.7643	0.95	[0.88, 1.03]	0.2318	0.94	[0.89, 1.00]	0.0345
MVPA (hour)	Set 1	1.15	[0.78, 1.67]	0.4823	1.13	[0.70, 1.83]	0.6065	0.96	[0.65, 1.51]	0.9636
	Set 2	1.18	[0.81, 1.73]	0.3811	1.01	[0.63, 1.63]	0.9626	0.85	[0.56, 1.30]	0.4584
	Set 3	1.30	[0.88, 1.90]	0.1853	1.16	[0.72, 1.88]	0.5461	0.89	[0.58, 1.37]	0.6104
VPA (minute)	Set 1	0.97	[0.91, 1.04]	0.4082	0.99	[0.91, 1.08]	0.8102	1.02	[0.94, 1.10]	0.6610

	Set 2	0.97	[0.91, 1.04]	0.3768	0.98	[0.90, 1.07]	0.6812	1.01	[0.94, 1.10]	0.7587
	Set 3	0.97	[0.91, 1.04]	0.4210	0.99	[0.91, 1.08]	0.7906	1.02	[0.94, 1.10]	0.6926

SAM: Substitute Afternoon for Morning
SAE: Substitute Afternoon for Evening
SME: Substitute Morning for Evening

[1] This means increasing PA in the afternoon by one unit while decreasing PA in the morning by one unit.
[2] The first set was adjusted for basic information: sex, age, ethnicity and BMI. The second set was added adjustment for socioeconomic factors: education and household income. The third set was further adjusted for lifestyle factors: smoke status, alcohol intake, sleep duration, and dietary information.

ESM Figure 1. Box plot of MVPA (measured in hours per day) stratified by timing and T2D outcome.

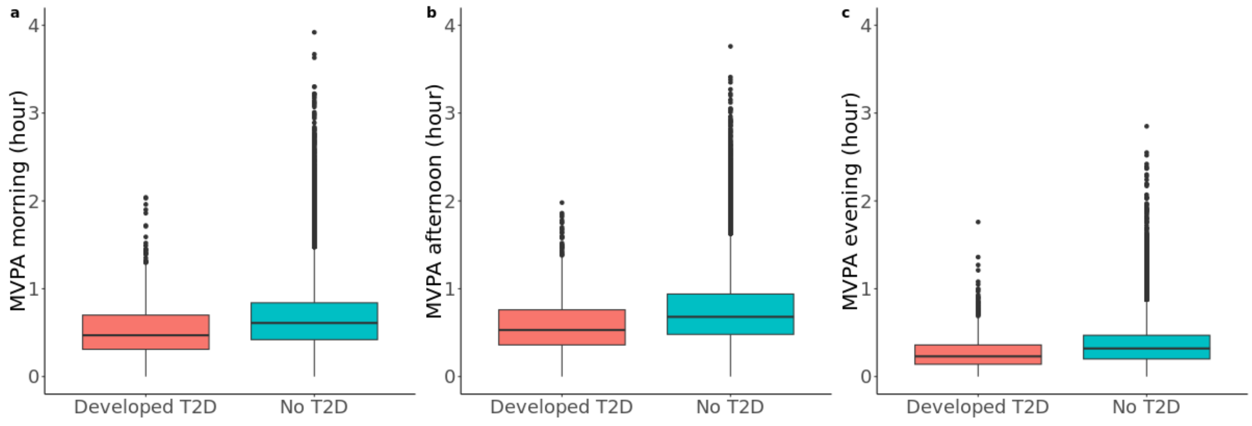

ESM Figure 2. Box plot of VPA (measured in minutes per day) stratified by timing and T2D outcome.

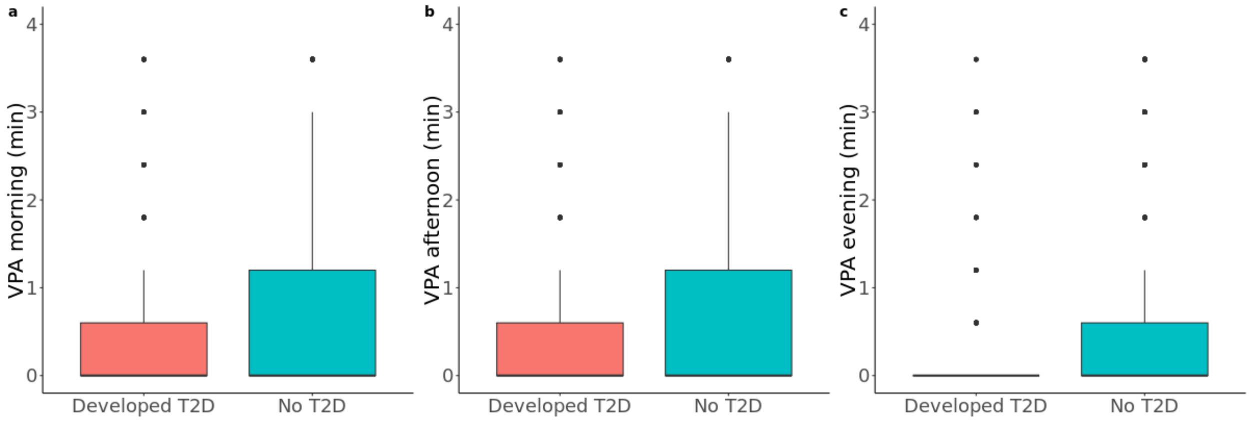

Supplement: Supplementary file 1 — Supplementary file1 (PDF 127 KB) [file 125_2023_6001_MOESM1_ESM.pdf]
